# From fish tank to bedside in cancer therapy: an interview with Leonard Zon

**DOI:** 10.1242/dmm.016642

**Published:** 2014-07

## Abstract

Leonard Zon, who is based at Harvard Medical School, is internationally recognized for his pioneering work in hematology and stem cell biology. His lab uses zebrafish as a model to understand blood cell development and, in recent years, has made inspiring breakthroughs in the treatment of blood diseases and cancer, helping to establish zebrafish as a powerful model for translational research. In this interview, Leonard speaks to *Disease Models & Mechanisms* Editor-in-Chief, Ross Cagan, about the evolution of his career from developmental biologist to physician-scientist and the stories behind some of his major research accomplishments. He also discusses challenges and opportunities in zebrafish research and provides advice on translating basic research findings to the clinic.

Leonard (Len) I. Zon was born in Hartford, CT, USA. He received his undergraduate degree in Chemistry and Natural Sciences from Muhlenberg College in Allentown, PA and, in 1983, obtained an M.D. degree from Jefferson Medical College in neighboring city Philadelphia. While undertaking clinical training at Deaconess Medical Center, he was appointed a fellow in medical oncology at Dana-Farber Cancer Institute. As a post-doctoral research associate in Stuart Orkin’s lab, Len sought to understand how specific blood lineages are programmed at the molecular level. During this time, he cloned *GATA-1*, which encodes a transcription factor that is essential for hematopoiesis. Despite having made significant breakthroughs in HIV research as part of his clinical training, this finding inspired Len to continue working at the bench to investigate blood cell development using animal models. This decision ultimately led to his introduction to zebrafish as a model for vertebrate development and disease, and, although he began as an outsider to the field, he has since established himself a leading figure in the zebrafish world. The Zon laboratory was one of the first to demonstrate the translational potential of the model, and Len has become an advocate for adopting zebrafish in drug discovery pipelines. As well as his notable successes in understanding and treating cancer, he is widely renowned for his work in stem cell biology. Len is currently the Grousbeck Professor of Pediatric Medicine at Harvard Medical School, Investigator at Howard Hughes Medical Institute, and Director of the Stem Cell Program at the Children’s Hospital Boston. He is founder of the International Society of Stem Cell Research (ISSCR), and recently co-founded the Zebrafish Disease Models Society.

**Figure:**
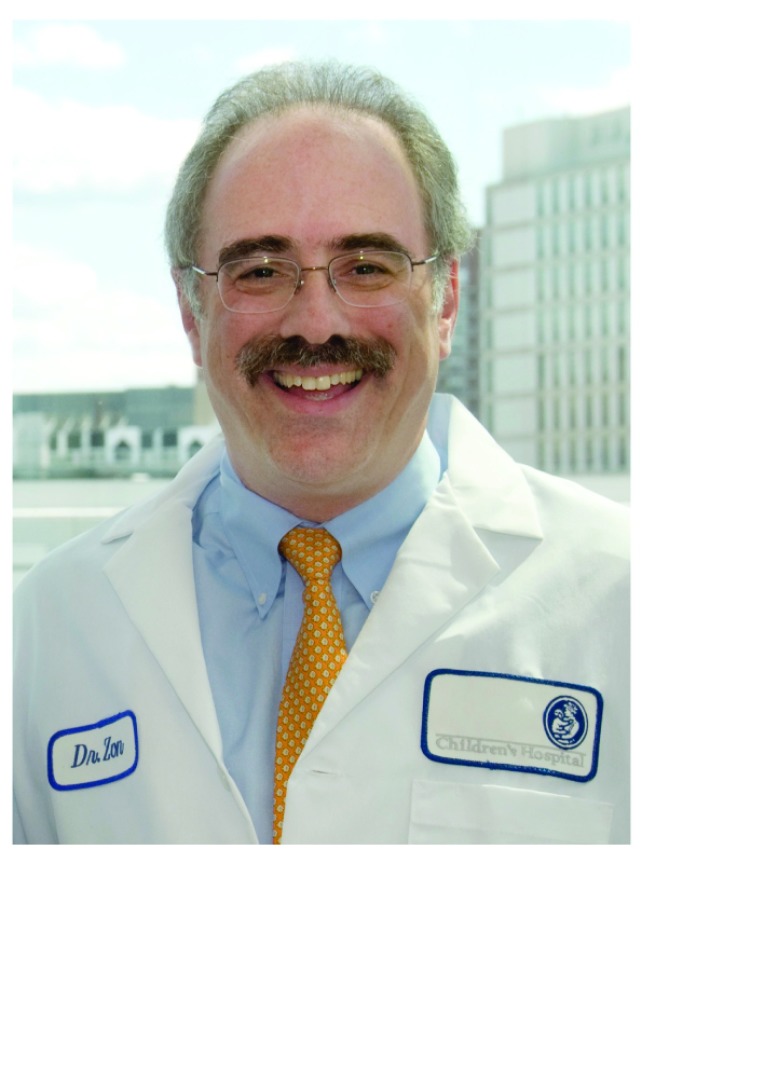


**Let’s start with your background. Why did you end up becoming a physician and also a scientist?**

The evolution of my career started while I was in college. I was doing research just to try it out but I didn’t really think that it was going to be for me – I really wanted to be a clinician. I decided I wanted to be a community pediatrician and put up a shingle and take care of patients. So, I applied for medical school [Jefferson Medical College]. At Jefferson, I went to a lecture by a famous hematologist, Allan Erslev. He had discovered a hormone called erythropoietin that stimulated the red blood cells to divide. I was so impressed that a hormone could control blood cell development that I asked if I could work in his laboratory during the summer. Once I was in his lab I started perfusing rat livers in search of erythropoietin and it’s then that I decided this was the career for me. Allan advised me to go to Boston to become an academic hematologist, and so I ended up doing my clinical training at Harvard. Around the same time the AIDS epidemic was in full swing, and I started looking with interest at blood counts of AIDS patients. I was one of the first people to note that individuals with AIDS are anemic and have a low platelet count. This discovery resulted in a *Nature* paper, and I became even more hooked on research.

Soon afterwards, I was appointed as fellow at the Dana-Farber Cancer Institute. I think most people expected that I would end up as a clinician, but I felt that I needed to immerse myself in basic research. I had always wanted to go to Stuart Orkin’s lab, and luckily I was able to get a post-doc position in his group. This move started me on the track to becoming a research scientist. It turned out I was good at basic science research and I really enjoyed it and got great mentorship in Stuart’s lab.

**It’s interesting that you have worn the two hats of ‘clinician’ and ‘basic scientist’ since the early days. Which people influenced you the most at the start of your career?**

The first person was Allan Erslev, who I’ve already mentioned. There was also an assistant professor at the time, Jaime Caro, who really mentored me in my first lab experience. Allan has unfortunately passed away, but I still talk to Jaime about things.

I think you couldn’t pick a better mentor than Stuart [Orkin]. He is also a physician as well as a scientist, and he applies an extreme rigor to his science – it is always high quality. He shoots for the stars but always maintains this incredible rigor in his work, which has taught me a lot about how to do science. I’ve been lucky enough to have the office next door to him for 20 years and it’s been wonderful.

I have a few other mentors, including Sam [Samuel] Lux, who is a specialist in hematology. He is the person who discovered ankyrin over in Harvey Lodish’s lab. Sam is an excellent scientist, a very smart person, and wonderful to talk to and approach for advice. There’s also David Nathan, who is the kingpin of hematology – he was chairman of pediatrics, chairman of hematology and is also the president of Dana-Farber. He has given me a lot of great advice over the years. Lastly, one of my mentors was Jerry [Jerome] Groopman, who is a HIV researcher. I worked with him during my medical residency, looking at the blood manifestations of AIDS. He now writes a lot in *The New York Times* and has written books on patient care.

“At the top of my list of messages to pass on to trainees is how to be a good scientist. I think a big part of this is being able to take risks”

**What did you learn from these mentors in terms of ways to run a lab? Some excellent scientists have come out of your group – what’s your style of training them?**

I have a very big group, currently with 19 post-docs and ten students. It is probably one of the larger laboratories in the institute. I try to treat people the way I would want to be treated and pass on advice that I think will be the most useful. At the top of my list of messages to pass on to trainees is how to be a good scientist. I think a big part of this is being able to take risks. When starting out, a lot of people like to do a safe project that is likely to finish in 2 years, and this may suffice for a short period of time, but later on when you’re trying to get your own lab going, you need to have breadth and to take risks. I try to help students and post-docs to have a balanced portfolio, so most members of my lab have two projects: a 75% project that aims to change the world and a 25% project that is almost guaranteed to work.

Doing the science right is the top priority, but, for running a lab, interpersonal skills are also important. Early career researchers aren’t given much formal training on how to run a lab and how to treat people. There is a mentoring system in place for my graduate students – I meet with them every other week and make sure I’m imparting advice on how to do science. I also have regular meetings with post-docs and, over the past 3 years or so, I’ve been coordinating talks where I cover topics that are not scientific but give tips on how to run a lab; for example, how to manage a budget, and how to market yourself. These are things I think scientists need to know. As a PI, you can’t meet everyone’s needs in a lab, although you hope that you do. Some people end up doing really well and some people sadly don’t, but you just do the best you can as a mentor.

“Doing the science right is the top priority, but, for running a lab, interpersonal skills are also important”

**Moving back to your research – how did you get involved with zebrafish? It’s quite a leap for someone who was used to seeing patients.**

To tell this story I need to go back to the point where I had just cloned the gene encoding a transcription factor called GATA-1 in Stuart’s lab. This is the master regulator of the red blood cell lineage. The interesting thing about GATA-1 is that stem cells don’t really express it – it’s red-blood-cell-specific. As I started to set up my own lab, I wanted to figure out how the transcription factor was being turned on. I knew I didn’t want to just define the regulatory elements in the *GATA-1* promoter and so I started to think about it as a developmental problem. How does an embryo turn this gene, and thereby blood development, on? I decided to use mouse genetics to get some answers. So, I went over to see a friend at MIT to get help on dissecting out 7.5-day-old mouse embryos – this is when the yolk-sac blood island originates. After 6 hours, I had only six embryos in a dish, and I knew that the low number of embryos would make everything I thought I could do impossible. It was very frustrating.

When I returned to my lab, a friend of mine called Celeste Simon who is now a Professor at Penn [University of Pennsylvania] happened to be there, and she said I looked terrible. I told her what had happened, and she invited me to a party at her house. It was at this party that I first came across Gerald Thomsen, who is now at Stonybrook, and I shared my story about dissecting mouse embryos with him. He said I needed an externally fertilized animal like *Xenopus*. An externally fertilized embryo can be easily observed, and eventually blood formation can be visualized. Gerry was working in Douglas Melton’s lab at the time, and he suggested that I go over and have a chat with Doug about using frogs to study hematopoiesis. With Doug’s help, things started to take off and after 2–3 years we were able to inject a homeobox gene into a frog embryo and turn the entire embryo into a blood island. This was very exciting, but I still wasn’t sure I wanted to stay in the frog system – it’s difficult to do genetics in frogs, and I always valued genetics.

Around that time, I presented the frog work at a hemoglobin-switching meeting off the coast of Seattle. Frank Grosveld [now at Erasmus MC], who works on transgenic mice, was there and he wanted to talk to me about my presentation. He said he loved what we’d done with frogs, but felt it wasn’t the right system to do genetics, and he pointed me towards zebrafish. He made a big point that if you immerse yourself in a field early on then you will really reap the benefits later on, as he had done with transgenic mice. Anyway, I thought about it, and the next day I was contacted by an investigator named William Dietrich, who is at Northeastern University. Bill works on the Antarctic icefish. These fish have lost the expression of globins during evolution, as an adaptation to their environment in Antarctica. Bill was interested in coming to my lab on sabbatical to study the effect of cold adaptation on the transcription of globin genes. I said I didn’t want to work on Antarctic fish but asked if he was interested in working on blood development in zebrafish – and he was. We got hold of a zebrafish mutant with no blood from Walter Gilbert’s lab and, within a week, people were saying to me: “you’re going to be a zebrafish researcher”. And that’s what happened.

**You started as an outsider but quickly became an insider in the zebrafish field. How have you seen the field change?**

The field was kind of set up by an amazing group in Oregon who focused on the neurobiology of fish. Other groups started to establish screens to look for mutants that would affect organ development. Then there was an influx of people, including me, who were interested in disease. By the end of the last century there were a fair number of investigators all working on screening mutant fish to understand organ development, with most of the work being done on embryos. It was an exciting time and we learned a lot.

The technology took off after the year 2000. Morpholinos were developed and we could finally do genetics, and use it to find out which pathways affect which organs. In my lab, we were looking for mutations that decrease blood cell levels, and four times we identified a mutant where the gene hadn’t previously been characterized. Then we found humans who carried mutations in those genes, so effectively discovered four novel human diseases as a result of research in fish.

What I’ve really seen burst onto the scene is transgenics. At a certain time I remember trying to convince my lab to create a transgenic zebrafish mutant and I couldn’t get anybody in the lab to do it. Then Shuo Lin published a paper showing the *Gata-1* promoter driving GFP and showed beautiful images of fluorescently tagged blood cells circulating. The imaging provides an unbelievable amount of information about biology. To see GFP-positive blood cells in circulation was amazing.

In the late 2000s, chemical biology screens took off. I think you could easily argue that zebrafish is the best chemical system. Access to the embryos is so easy, and the field really blossomed with interesting screens into all sorts of phenotypes. Now I think we are at the beginning of another huge revolution with CRISPR technology. Using this technology, my lab was able to make a mutant in just 2 months – it was incredible. With a lot of groups interested in disease combined with powerful genetics, we’re inevitably going to see a growth in zebrafish disease models. In fact, a group of zebrafish researchers (Liz Patton, Jim Amatruda, Jill DeJong and I) recently launched the Zebrafish Disease Models Society and we see this as an increasingly important part of the zebrafish community.

“I think you could easily argue that zebrafish is the best chemical system. Access to the embryos is so easy, and the field really blossomed with interesting screens into all sorts of phenotypes”

**You’ve mentioned that you started as a developmental biologist and you have walked forward from this looking at the links between blood, stem cells and disease. Where do you think this can go in terms of therapeutics?**

I think the zebrafish works very nicely as a translational model. So far, we have had two success stories of clinical translation – from the tank to the bedside. In the first, we found a small molecule that can amplify blood stem cells in a zebrafish embryo. Being a hematologist, I knew immediately what to do with this. I presented the finding at Dana-Farber, where I had trained and still had contacts, and it was taken rather seriously. We were given the opportunity to work with the Center for Human Cell Therapy, which is geared to take basic discoveries and use them to develop standard operating procedures that could be put into the clinic. By this time, we had been able to show that the prostaglandin chemical we’d identified could be added to blood stem cells in a mouse and could amplify the number about fourfold. We recognized that the clinical situation that it was needed for was in patients who were getting a cord blood transplant to treat leukemia. They benefit from the cord blood transplant after chemotherapy because it reconstitutes their immune system, but a single cord blood transplant only allows an engraftment of about 60% of the patients. So, the standard of care now is to get cord bloods from two different donors. As these cord bloods have different genetic origins, they end up ‘fighting’ each other and eventually one of the cord bloods ‘wins’. The immunological fight that happens isn’t a pretty one and it would be better to avoid it by amplifying stem cells in cord blood from one source. To test this, we took human cord blood samples, split them and treated half with our small molecule (the other half were untreated), before putting them into immunodeficient mice. We showed that more mice ended up with human blood if pre-treated with the chemical. We then set up a clinical trial involving 12 leukemia patients who were given high-dose chemotherapy. They were going to be given the ‘standard of care’, which involves two cord bloods, but we pre-treated one of the cord bloods for 2 hours with our chemical and then gave both to the patient. In ten out of 12 patients, the treated cord was the one that preferentially engrafted, and the neutrophils and platelets came back earlier after these transplants than in control patients who had not been treated with PGE2. This has gone from a Phase I trial now into Phase II, where we will treat about 50 patients.

The second story is relevant to melanoma. The melanocytes of your skin are derived from the neural crest. Neural crest cells also make other tissues, like the Schwann cells or cartilage. We wanted to find a melanoma drug and we thought about antagonizing the neural crest. We screened in embryos for a chemical that could erase the neural crest and, out of 3000 small molecules, only one did this. This was a novel chemical but we were able to use a chemoinformatics database to find out that it inhibits an enzyme called DHODH, which catalyzes a step in pyrimidine biosynthesis. There is a drug on the market, used to treat rheumatoid arthritis, that also targets this particular enzyme, and we were able to show that this other chemical, leflunomide, causes the same phenotype in our screen. In a series of experiments, we showed that leflunomide blocks the transcription of neural crest genes and also some proliferation genes. We then showed in human tumor xenografts in mice that leflunomide is effective as an anti-melanoma drug, and confirmed that leflunomide pauses transcription of neural crest and proliferative genes in human melanomas. We’ve now treated three patients in a clinical trial with leflunomide (together with a BRAF inhibitor that also attacks cell proliferation) and we’re hoping to treat 43 patients in the next phase.

An important thing that these experiences have taught me is that it’s possible to put fish into a drug discovery pipeline but you’re going to need multiple model organisms to actually be able to translate. It’s pretty hard to go straight from the fish into humans so we had to rely on intermediate steps involving mice. This is likely to be a paradigm for how to translate work from the zebrafish.

“An important thing that these experiences have taught me is that it’s possible to put fish into a drug discovery pipeline but you’re going to need multiple model organisms to actually be able to translate”

**If you were to give advice to us non-physician basic researchers, what would it be?**

I hope that as people do their basic research they can try to project it somehow to the clinic. I feel that when I’m mentoring people at Harvard Stem Cell Institute for instance, sometimes there’s a desire to publish basic discoveries and never get to the translation part of it. I’ve been trying to show them there is a way to get your basic research to the clinic.

If you do have the desire to translate your work, then early mentoring is absolutely critical. It’s best to get in touch with a physician-scientist or medical doctor. I think it’s a good idea to present your ideas to a doctor and get their feedback. Sometimes you might have a basic discovery and you think that you’re moving in the right direction, but somebody who is taking care of patients might realize it’s impractical and there’s a better alternative. I will often say “go and present your work on clinical rounds”. You have to be thick-skinned but it can be very revealing. Meeting patients is also rewarding. We are starting to do that with PhD students through the Leder Human Biology and Translational Medicine program at Harvard Medical School.

**If I were a young scientist thinking about using zebrafish to study disease, I guess you would recommend it? What needs to improve to make the model even more useful?**

There are a couple of things I’m hoping for. A lot of people have started working on adult zebrafish. We now have adult fish that are transparent, like the embryos, and this opens up a lot of opportunities; for example, in my own lab we’re doing marrow transplants in adults. However, what we don’t have is a good way of characterizing adult physiology. Every assay that exists in a human can be applied in some way to the fish, but not much has been done. A major limitation is that we lack antibodies, which would speed things up and allow the field of adult biology to grow – I’d like to see more reagents and antibodies be developed to allow this.

I would also like the infrastructure of zebrafish research to be built up a little bit better than it is. For instance, the FlyBase is amazing and the *Drosophila* stock centers are well-developed. For fish, we have two wonderful stock centers that function well, but we don’t have much of an infrastructure to distribute and characterize the strains. The NIH and other funding organizations could help with this.

Another thing that I feel is lacking is a voice or influence to push the field forward, although this has improved since the early days. Most people worry about their own research questions and laboratories, and nobody has really pushed the buttons that need to be pushed to give zebrafish research a more public face. It was similar in the stem cell field, until investigators including myself stepped forward to gain more support from different bodies, governments and the public. There are lots of great meetings and great science in the zebrafish world, but I don’t think the field is reaching its potential. Funding is low in general, and I feel that now we’re doing so many fantastic things – modeling disease and coming up with new therapies – we need to start blowing our horn more to catch the attention of funding bodies. This is why I think the Zebrafish Disease Models Society we’ve started will be important.

**You have a physician hat, a researcher hat, and you’ve also collaborated with pharmaceutical companies. How useful have you found your interactions with companies?**

I would encourage anybody who is starting out their lab to start thinking about interacting with companies as early as possible. When I initially started my lab, we were awarded a grant by Hoffmann La-Roche, which meant we could take yearly trips to them to learn more about how pharmaceutical companies do their work. It was really eye-opening, particularly in terms of the scale of the operation, to see how pharma does basic science. Some of the people who I befriended there ended up working at Novartis, and they asked me to be on the functional genomics board for Novartis. Their meetings were incredibly intense, and gave me an insight into what decisions and processes a major pharma company has to go through. When I become a PI, I took on a consultant role for several start-up companies. Being on the scientific advisory board of these companies, I could really see how they matured and what determined success or failure.

“I would encourage anybody who is starting out their lab to start thinking about interacting with companies as early as possible”

In 2007 or so, a group of my friends on the west coast were approached by venture capitalists to start a company. They had interesting intellectual property associated with their laboratories but they wanted to have some input from physician-scientists. I was brought in as one such person, as well as David Scadden [Massachusetts General Hospital], and we became the founders of a new company called Fate Therapeutics. Having been on the scientific advisory boards of companies I knew a fair amount about the process, but being a founder is fundamentally different. It was interesting to see how the intellectual property transfers to a company and how patents are processed. The interactions with the company were extremely useful, and in fact I think these were absolutely critical to the success of my lab’s prostaglandin project for amplifying stem cells. The company, which has about 45 employees, went public at the end of 2013 and seems to be doing well. Together with a friend, I’ve just started another company, which is called Scholar Rock.

There’s a rich biology on how to produce a drug on the basis of a basic research finding, and it’s really great for scientists to learn about this. Interacting with companies can be very fruitful.

**If you hadn’t gone for science, what career path might you have taken?**

I would have been a professional trumpet player. I played in high school and I was part of an intercollegiate band and orchestra while in college. I have played for 32 years in the same symphony orchestra here at Harvard. I really enjoy playing the trumpet and I come from a musical family – my brother was a child prodigy on the piano, and now studies music at Durham University in England.

